# Nutrition Education Among Community-Dwelling Polish Seniors—A Pilot Study of Diet Quality, Health Status, and Public Health Interventions

**DOI:** 10.3390/nu17132103

**Published:** 2025-06-25

**Authors:** Anna Szreiter, Sabina Lachowicz-Wiśniewska

**Affiliations:** Faculty of Medicine and Health Science, University of Kalisz, W. Bogusławskiego Square 2, 62-800 Kalisz, Poland; 29956@uniwersytetkaliski.edu.pl

**Keywords:** population aging, older adults, nutrition, domino effect, pHDI-10, questionnaire, diet quality

## Abstract

Background: Population aging presents major public health challenges. Nutrition education has emerged as a key intervention to improve diet quality and reduce the risk of chronic diseases among older adults. Methods: This pilot cross-sectional study assessed the effects of a brief nutrition education session on dietary patterns, lifestyle behaviors, and health perceptions among 151 community-dwelling Polish seniors aged 60 and over. Data were collected using the KomPAN® questionnaire, the Pro-Healthy Diet Index (pHDI-10), the Non-Healthy Diet Index (nHDI-14), and self-reported health indicators. Results: The findings revealed suboptimal dietary patterns, including low consumption of whole grains, legumes, and fish. A high prevalence of chronic diseases was observed, particularly hypercholesterolemia (67.7%) and hypertension (53.1%). A weak but significant correlation was found between BMI and the number of diagnosed conditions (r = 0.3, *p* = 0.003). Despite limited prior nutritional knowledge, participants perceived the educational session as beneficial, and many expressed an intention to share the acquired information with peers, indicating a potential “domino effect”. Conclusions: Although the sample size limits generalizability, the results support the effectiveness of brief, tailored nutrition education as a scalable, cost-effective public health strategy. Such interventions may promote healthy aging, reduce diet-related disease burden, and enhance peer-driven knowledge dissemination among older adults.

## 1. Introduction

Population aging is one of the most significant public health challenges of the 21st century. According to the World Health Organization, by 2050, the global number of individuals aged over 60 will double, creating mounting pressure on healthcare systems and increasing the need for effective preventive strategies [1]. One of the most important and modifiable determinants of healthy aging is nutrition. A well-balanced diet helps reduce the risk of non-communicable diseases, such as cardiovascular disease, type 2 diabetes, and osteoporosis, while also supporting functional independence and quality of life in later years [2,3,4].

In Poland, as in many developed countries, awareness of the relationship between nutrition and health is increasing; however, nutritional risk and malnutrition remain prevalent among seniors. Common deficiencies include inadequate intake of protein, omega-3 fatty acids, vitamins B6, B12, D, and E, and minerals, such as calcium, magnesium, and zinc [5]. Poor dietary practices, such as low vegetable and fruit intake and high consumption of salt and saturated fats, can exacerbate age-related decline in health status [1,6].

Nutrition education is a key intervention strategy for improving dietary behavior among older adults. Numerous studies have demonstrated that long-term education programs can enhance both dietary knowledge and food choices [7,8]. However, relatively few studies have investigated the short-term impact of such interventions, particularly in the context of seniors living independently in local communities.

Additionally, there is a lack of data on how acquired knowledge is shared beyond the individual level. Seniors may serve as informal health educators within their families and social networks. This process, referred to as the “domino effect”, may help promote healthy dietary practices at a community level, yet it remains underexplored in the scientific literature [9,10].

From an economic perspective, preventive measures such as nutrition education may also offer long-term healthcare savings. In 2023, Poland’s National Health Fund allocated 55.6% of its budget to healthcare services for individuals over 60, with geriatric hospitalizations and medication reimbursements constituting a major share. International analyses have suggested that targeted investments in nutrition interventions could prevent up to 1.6 million hospitalizations annually and save billions in healthcare spending [11,12,13,14]. By influencing peers or family members, seniors can reinforce positive behaviors in others, multiplying the benefits of a single educational effort. This phenomenon has been observed in health promotion networks.

Although many studies have documented the effectiveness of long-term nutrition education in older adults, relatively few have evaluated the short-term impact of such interventions, especially among seniors independently living in community settings. Moreover, limited attention has been given to the indirect effects of nutrition education, such as peer-to-peer knowledge transfer within social networks—the so-called “domino effect”. These gaps highlight the need for targeted pilot studies to assess the feasibility and potential benefits of brief, low-cost interventions adapted to the functional and cognitive capacities of seniors.

Therefore, the aim of this pilot study was to evaluate the short-term impact of a structured nutrition education session on diet quality, lifestyle behaviors, and health perceptions among community-dwelling older adults in Poland. Additionally, the study explored the potential for informal dissemination of acquired knowledge within participants’ social circles. The study also explored the potential for the informal dissemination of acquired knowledge within participants’ social circles. This aspect of the study was exploratory in nature and based on subjective self-reporting rather than formal behavioral tracking. While not formally measured, this phenomenon emerged from self-reported declarations and warrants further investigation.

## 2. Materials and Methods

### 2.1. Study Design and Participants

This cross-sectional observational study was conducted between October 2024 and February 2025 in the Greater Poland Voivodeship and several other regions across Poland. The study targeted individuals aged 60 years and older. A total of 151 participants were recruited through direct contact. The sample size (*n* = 151) was considered appropriate for a pilot study aiming to explore preliminary associations and inform the design of future large-scale studies. The recruitment took place at senior community centers, social welfare institutions, and individual households. Inclusion criteria involved age (≥60), informed consent, and the ability to participate in a structured interview.

The research protocol was approved by the Bioethics Committee of the University of Kalisz (Approval no. KB-551/2024), and the study complied with the principles outlined in the Declaration of Helsinki [15]. The study was conducted in the Greater Poland Voivodeship and additional regions, including Łódź, Kuyavian–Pomeranian, Lower Silesian, Opole, Silesian, Masovian, Pomeranian, Warmian–Masurian, West Pomeranian, and Lubusz. Residence was classified per national statistics as urban (>100,000), small town (<100,000), or rural. All participants were informed about the voluntary and anonymous nature of their participation and signed informed consent forms before data collection. Participants were recruited by trained research personnel in cooperation with senior center staff. The study was performed using a paper-based version of the KomPAN^®^ Questionnaire [15,16,17] developed by the Committee of Human Nutrition Science of the Polish Academy of Sciences. Exclusion criteria included cognitive impairment, inability to provide informed consent, or lack of availability for full questionnaire participation based on subjective assessment.

Due to these methodological features and contextual constraints, the key limitations of this study are addressed in Section 4.

### 2.2. Research Tool and Data Collection

The study employed a structured interview method using a custom-designed questionnaire composed of 67 items. Prior to questionnaire administration, participants received a brief nutrition education session based on the latest dietary recommendations for older adults developed by the National Institute of Public Health–National Institute of Hygiene (NIZP-PZH, Poland), which align with international guidelines, such as those from the WHO and EFSA [17]. This pre-survey intervention was intended to increase participants’ nutritional awareness and ensure a better understanding of the content and context of the questionnaire. The questionnaire assessed socio-demographic data (e.g., age, gender, education, and household composition), health status, and diagnosed chronic conditions; physical activity and lifestyle habits; dietary patterns and eating behavior; knowledge and attitudes regarding nutrition, supplementation practices, and medication use; and opinions on dietary education and its role in health promotion.

The KomPAN^®^ questionnaire was developed and validated by the Polish Academy of Sciences for use in adult populations and has been successfully applied in previous studies involving older adults in Poland [18]. To ensure clarity and appropriateness for senior participants, the paper version was administered in the form of a structured interview, allowing trained researchers to explain questions if necessary and minimize comprehension-related issues.

The questionnaire included both closed and semi-open questions, and responses were recorded during one-on-one interviews by trained research assistants.

Diet quality was assessed using the pro-healthy diet index (pHDI-10), which evaluates the frequency of consumption of ten food groups with established health benefits. They also assessed the Non-Healthy Diet Index (nHDI-14), which included consuming 14 food groups considered detrimental to health, such as white bread, fast food, red and processed meats, sweetened beverages, and lard. Scores were calculated based on validated scoring procedures, as outlined by Gajda et al. [19] and the KomPAN^®^ questionnaire [16], and expressed intake frequency as times/day. In addition, Body Mass Index (BMI) was calculated using self-reported weight and height. A correlational analysis between BMI and the number of chronic diseases was performed.

Based on the calculations performed according to the procedure described by Gajda et al. [19], three levels of diet quality indices were identified: three levels representing the intensity of adherence to dietary recommendations within the pro-healthy diet index (pHDI-10: moderate, low, and high) and two levels within the non-healthy diet index (nHDI-14: low). The “moderate” level in nHDI-14 was not observed among the participants. Due to the variability in the frequency of consumption of foods included in both indices, six dietary profiles were developed to provide a more comprehensive characterization of participants’ diet quality. These profiles resulted from the combination of pHDI-10 and nHDI-14 values, as shown in Table 1.

All analyses were performed using fully completed questionnaires. Forms with incomplete responses were excluded prior to analysis; therefore, no missing data were present in the final dataset.

### 2.3. Statistical Analysis

The following categorical variables were included in the analysis: gender, age group, place of residence, education, and profile of diet quality index. Categorical data were presented as frequencies (N) and percentages (%). The chi-square test was used to assess significant differences between categories. Diet quality indices were calculated according to the procedure previously described by Gajda et al. [19]. The validity of the index-based variables was confirmed using the Kaiser–Meyer–Olkin measure of sampling adequacy and Bartlett’s test of sphericity. Both tests yielded statistically significant results. The KMO value was 0.768, while Bartlett’s test was significant at *p* < 0.0001. A *p*-value < 0.05 was considered statistically significant for all tests. Statistical analyses were conducted using STATISTICA software (version 13.3 PL; StatSoft Inc., Tulsa, OK, USA; StatSoft, Kraków, Poland). Descriptive statistics (means and standard deviations) were calculated for BMI and the number of chronic conditions across various demographic and behavioral categories. A Pearson correlation test revealed a statistically significant positive relationship between BMI and the number of diagnosed chronic diseases (r = 0.297, *p* = 0.003), suggesting that higher BMI values were associated with more comorbidities. To examine differences in BMI and morbidity by level of physical activity, a non-parametric Kruskal–Wallis test was performed. The test showed no statistically significant differences in BMI (H = 0.86, *p* = 0.648) nor the number of chronic diseases (H = 3.13, *p* = 0.201) across groups reporting varying levels of physical activity. Additionally, a Mann–Whitney U test comparing BMI between physically active and inactive respondents showed no significant difference (U = 978.5, *p* = 0.4499), although the average BMI was slightly lower in the physically active group (28.45 vs. 28.71). The strength of association for categorical variables was assessed using Cramér’s V, with the following interpretation: values below 0.10 indicate no or very weak association, 0.10–0.20 indicate a weak association, 0.20–0.40 indicate a moderate association, 0.40–0.60 indicate a strong association, and values above 0.60 are considered very strong, though rarely observed.

## 3. Results

### 3.1. Sociodemographic Profile and BMI Classification

An analysis of the age distribution by gender showed that the largest group of respondents was women aged 66–70 years, accounting for 45.2% of all female participants. Among men, the same age group also predominated, representing 35.3% of the male study population (Table 2).

In terms of education level, most respondents (62.5%) reported having completed secondary education. Only 3.1% had attained primary education, 12.5% had vocational training, and 21.9% reported holding higher education degrees.

Regarding marital status, the majority (55.2%) were married or in a partnership. Widows and widowers accounted for 25%, 15.6% were single, and 4.2% selected “other”, reflecting the sensitive nature of the question and the characteristics of the surveyed group.

The largest number of respondents lived in the Greater Poland Voivodeship (*n* = 50), with others residing across ten additional regions. Based on classification by residence type, 42.7% lived in small towns, 33.3% in large cities, and 23% in rural areas.

More than half of the respondents (55.2%) lived in two-person households. A total of 24 participants lived alone, while smaller numbers reported living with three or more people. Only 1% lived with other seniors, and 13.5% reported living with children or in multigenerational households.

The majority of participants (*n* = 66) were retired or not professionally active. In terms of economic self-assessment, 39.6% rated their situation as good, 14.6% as very good, and only 2.1% described it as very modest.

Body Mass Index (BMI) was calculated based on self-reported height and weight (Table 2). No cases of undernutrition were observed among men, while 4.8% of women aged 60–65 were undernourished. The highest obesity rate was recorded in the 66–70 age group, with obese men accounting for 23.5% and women for 14.5%. A Pearson correlation coefficient of 0.3 indicates a weak positive correlation between BMI and the number of diagnosed chronic conditions [20,21].

### 3.2. Lifestyle Patterns, Health Perception, and Physical Activity

Retirement typically brings significant lifestyle changes, as older adults gain more discretionary time previously devoted to work. Despite reaching retirement age, some seniors remain professionally active, often due to economic constraints or personal circumstances.

**Figure 1:**
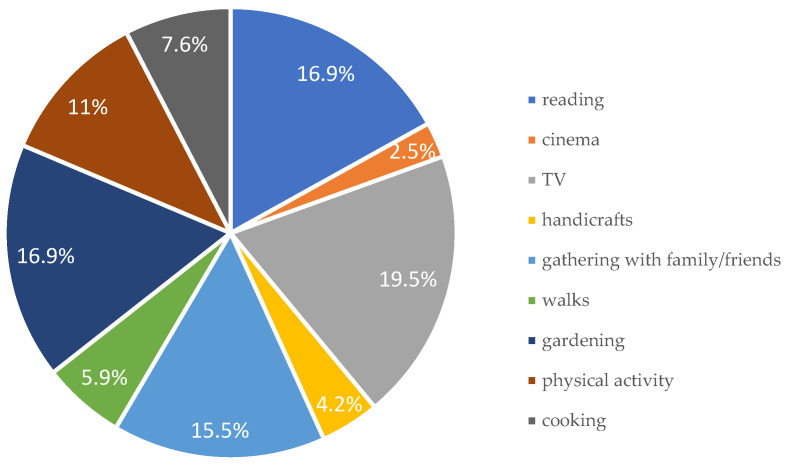
Declared ways of spending free time.

Only 26% of participants reported active involvement in a senior club. However, a large proportion (72.9%) used the Internet in their free time, accessing it through mobile phones, tablets, or computers. The most commonly reported leisure activities included watching television, reading, socializing with family and friends, and gardening (Figure 1).

**Figure 2:**
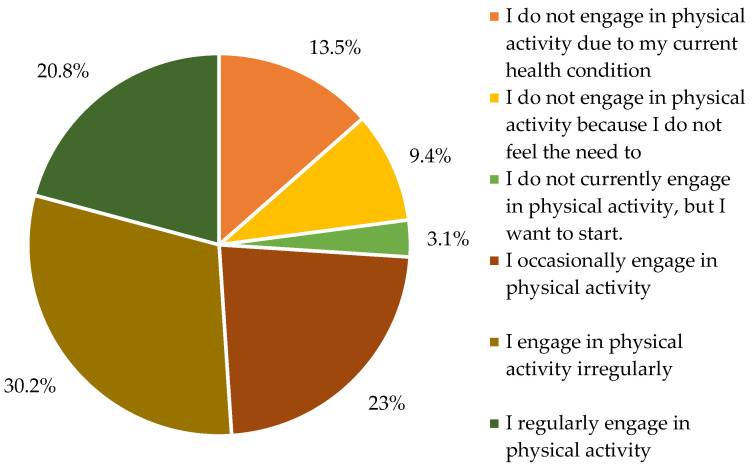
Frequency of physical activity among participants.

Regarding physical activity, 20.8% of respondents reported regular participation, 22.9% engaged occasionally, and 30.2% participated irregularly. Meanwhile, 26% of seniors stated that they did not engage in any physical activity. The most frequently cited barrier was current health conditions, followed by lack of motivation, limited access to senior-specific programs, financial constraints, and lack of time (Figure 2).

In terms of substance use, 88 respondents reported that they did not smoke, and only 8.3% declared regular tobacco use. Occasional alcohol consumption was reported by 91.7% of participants, while 8.3% consumed alcohol several times per week (Figure S1).

The majority of participants (70.8%) stated that they neither felt old nor young in relation to aging. Regarding stress associated with aging, approximately one-third reported no such experiences, while 29.2% acknowledged strong stress that negatively affected their daily functioning and overall health. A Pearson correlation analysis was performed to examine the relationship between perceived stress related to aging and the self-assessed health status of older adults. The correlation coefficient was r = 0.63, indicating a strong positive association between the examined variables [21].

**Figure 3:**
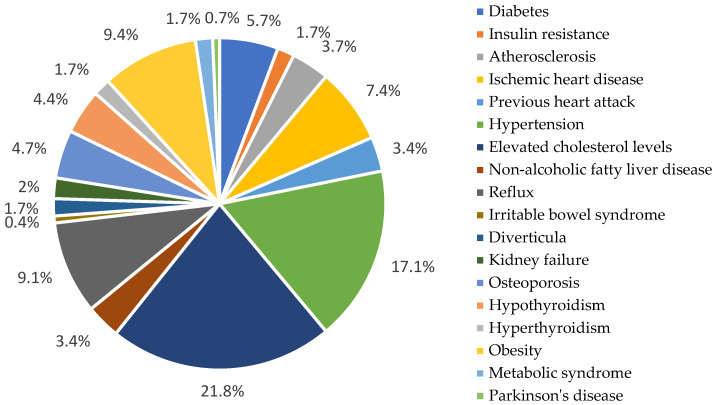
Declared disease entities of respondents.

The assessment of health status also included questions regarding disability, the number of diagnosed chronic conditions, and the use of medications and/or supplements (Figure 3). According to the data, over 70% of respondents did not have a formal disability certification. Nevertheless, when asked about the number of diagnosed chronic diseases, a total of 298 conditions were reported across all participants, averaging 3.1 diagnoses per person.

The most commonly reported conditions were elevated cholesterol and hypertension. According to the National Center for Nutrition Education, based on numerous long-term observational and clinical studies, elevated serum cholesterol levels—especially LDL cholesterol, commonly referred to as “bad cholesterol”—are associated with an increased risk of cardiovascular diseases [22]. Respondents reported a total of 298 diagnosed chronic conditions, averaging 3.1 diseases per person. The most frequently mentioned conditions included elevated cholesterol and hypertension. Over 70% did not have formal disability certification. Regarding supplementation, respondents most commonly used magnesium, vitamins C and B12, calcium, potassium, multivitamins, cod liver oil, dietary fiber, and herbal preparations. Daily vitamin D supplementation (1000–4000 IU) was reported by 29.2% of seniors, while 33.3% used it seasonally.

### 3.3. Dietary Habits, Meal Frequency, and Diet Quality Index (pHDI/nHDI)

**Figure 4:**
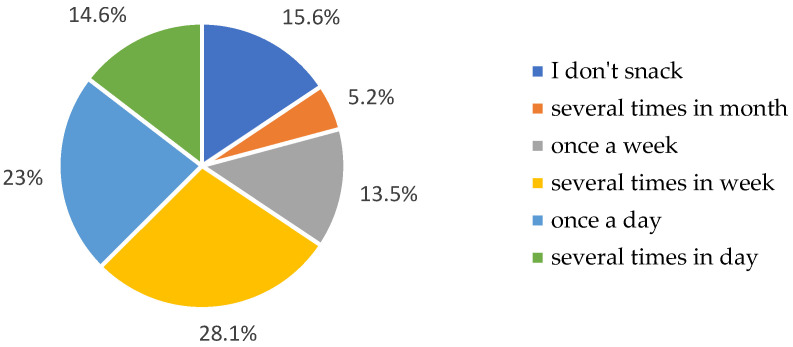
Frequency of snacking between meals chosen by participants.

**Figure 5:**
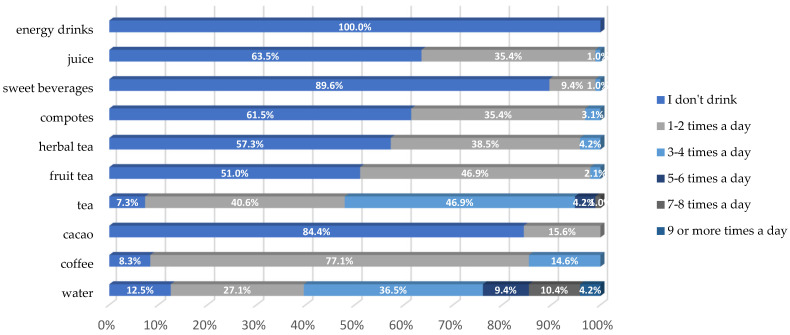
Types of snacks and frequency chosen by participants.

In response to the question regarding dietary practices, 87.5% of participants indicated that they were not following any specific diet. At the same time, 12.5% declared adherence to dietary regimes, such as diabetic, light/low-residue diets, intermittent fasting, or meal plan subscriptions. Regarding meal frequency, most older adults reported consuming three meals per day. However, only 18.8% indicated regular meal timing, while 55.5% followed a partially consistent schedule, as summarized in Table S1. The most frequently reported setting for eating meals was the home (Figure S2). As shown in Supplementary Figure S2, most meals were consumed at home, indicating a preference for domestic eating environments. When asked about snacking between meals, the responses ranked as follows: several times per week > several times per day > once daily > once per week or not at all (Figure 4). Among the most commonly chosen snack groups were sweet snacks > fruits > nuts > unsweetened/lightly sweetened dairy beverages and desserts > vegetables > salty snacks > sweetened beverages and milk-based desserts (Figure 5 and Figure S1). Figure 5 illustrates that sweet snacks were the most frequently chosen, followed by fruits and nuts. Supplementary Figure S3 provides a detailed frequency breakdown for other snack categories.

**Figure 6:**
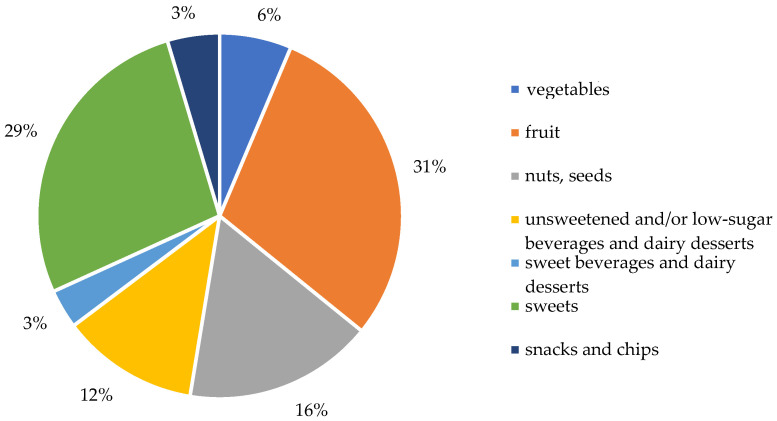
Types of snacks chosen by participants.

The survey also revealed that older adult respondents do not consume energy drinks, in contrast to younger individuals, who report high consumption levels of such beverages. Additionally, more than half of the older participants indicated that they do not sweeten their drinks. Among the preferred beverages selected by seniors were water, coffee, and tea (Figure 6).

The surveyed older adults reported egg consumption, with 40.6% stating they consume 3–4 eggs per week. Additionally, 58.3% of respondents indicated that they consume semi-skimmed dairy products, and 88.5% reported eating fish. The most commonly selected forms of fish included smoked, fried, baked, and canned varieties. Figure S4 shows the frequency of fermented dairy product intake, including natural yogurt, kefir, and cottage cheese, while Figure S5 presents consumption levels of other animal-based products, such as milk and cheese.

A total of 65% of respondents declared not consuming brown rice, while 58% stated they do not eat whole-grain pasta, and 28% reported not consuming oatmeal. Notably, fewer than one-quarter of the surveyed older adults consume wholemeal bread a few times a week. Only 14% of individuals reported consuming wholemeal bread once daily, and merely four respondents indicated consuming it several times a day. Regarding meat and meat product intake, pork was the most commonly consumed type, eaten a few times a week. In contrast, when it comes to lean poultry, 28% of respondents reported eating it once a week, and 27% consumed it several times a week. Ham was the most frequently selected meat product, as declared by 33% of respondents. A significant proportion of the older adult participants reported not consuming steak tartare or aged cured meats. Among pulses, the most frequently consumed types were beans > peas > broad beans > chickpeas and lentils > soybeans (Table S1).

Regarding thermal food processing, the most preferred cooking methods were boiling, stewing, and baking/frying with a minimal amount of fat, predominantly using vegetable oil. For spreading, 75% of respondents reported choosing butter as their preferred option (Table S1).

The consumption of ready-made foods, such as powdered soups, instant meals, or pre-packaged dishes, was declared as non-existent by 81.3% of the participants, while only 3.1% reported consuming them several times per week. As presented in Table S1, 43.8% of participants reported no consumption of fast food, while only 4.2% consumed it a few times per month.

Based on the reported food frequency data, the pro-healthy diet index (pHDI) and Non-Healthy Diet Index (nHDI) were calculated following established scoring criteria (Table 3). The mean pHDI-10 score was 3.57 (SD = 1.04; 95% CI: 3.44–3.70), while the mean nHDI-14 score was 2.70 (SD = 1.54; 95% CI: 2.56–2.83). These results indicate that 60% of the surveyed senior population followed diets with a low intensity of health-promoting dietary traits (L-QI). This result may be associated with relatively low and/or moderate intake of products included in the ten groups defined as beneficial to health. Moreover, 45% of respondents were characterized by a moderate-quality diet index (M-QI). This dietary pattern was predominantly observed among individuals living in urban areas with a partner or children, being professionally inactive, and holding higher education degrees. Only 16% of the study population demonstrated a high-quality diet index (H-QI), primarily among women residing in rural areas, living with a partner or children, professionally inactive, with higher education, and having a BMI within the normal range. (Table 3). Significant associations were found between the diet quality index (DQI) and several sociodemographic variables, including age, education, place of residence, residential status, employment activity, and BMI (all *p* < 0.05). The association with gender was not statistically significant. Based on Cramér’s V coefficients, the strongest relationships with diet quality were observed for education and age. Other variables, such as living situation, employment status, and place of residence, also showed moderate relationships with the DQI. All statistical outcomes are summarized in Table 3.

### 3.4. Seniors’ Views on Nutrition and Health Education

Most participants believed that regular meal consumption influences overall health. More than half also agreed that snacking between meals has an impact on health. When asked about the health effects of regularly adding salt to food, 33 respondents stated they had no opinion on the matter.

In their self-assessment, 46.9% of participants rated their dietary habits as neither good nor bad. Additionally, 44.8% described their eating behavior as good, while only 1% considered it very good. Conversely, 5.2% rated their habits as poor, and 2.1% as very poor.

When asked about their knowledge of healthy eating, most respondents described it as sufficient. However, only 10.4% of participants had ever consulted a dietitian or received professional dietary counseling.

Over half of the seniors agreed that nutrition education plays a role in health protection. A significant majority (89.6%) believed that nutrition education positively affects the health of older adults.

Participants identified several key barriers to healthy eating. The most frequently mentioned was having entrenched dietary habits (37.5%). Limited access to free nutrition education and insufficient knowledge were each reported by approximately 24% of respondents. A small percentage (4.2%) expressed fear of making dietary changes (Table S1).

## 4. Discussion

### 4.1. Lifestyle and Nutrition in Healthy Aging

The aging population poses increasing challenges to healthcare systems, both globally and in Poland. By 2050, the number of individuals aged 65 and older is projected to double to 1.5 billion worldwide [22,23,24,25,26,27,28]. This demographic shift is associated with gradual physiological decline and heightened vulnerability to chronic diseases and functional limitations. However, modifiable lifestyle factors—including balanced nutrition, regular physical activity, emotional stability, and adequate sleep—can significantly delay functional decline and improve quality of life [14].

Our study identified several lifestyle deficiencies among older adults, especially concerning physical activity. Only 20.8% of respondents reported engaging in regular physical activity, with the most frequently cited barrier being poor health (30.2%). This aligns with other studies showing low activity levels in older adults, particularly in rural areas with limited access to supportive infrastructure [29]. Tailored exercise programs, particularly progressive resistance training, have been shown to mitigate aging effects and reduce the risk of non-communicable diseases [30]. Physical activity can also reduce depression risk by 21%, and globally, physical inactivity contributes to 3.2 million deaths annually [31].

Emotional well-being also influences aging outcomes. We observed a strong correlation (r = 0.63) between perceived stress and self-assessed health, suggesting that psychological resilience plays a buffering role. Lifestyle patterns in later life—such as socially or physically active versus passive or home-centered styles—reflect whether emotional, cognitive, and social needs are met [32]. Active seniors tend to experience less loneliness, greater coping capacity, and higher life satisfaction [33]. In contrast to younger individuals, who report high consumption of energy drinks, older adults demonstrated complete avoidance of such beverages, potentially reflecting greater health awareness or differing lifestyle patterns.

Nutritional education plays a crucial role in improving lifestyle behaviors among older adults. Emphasis should be placed on promoting accessible physical activities like walking and improving hydration awareness. Education can help correct harmful habits, such as the insufficient intake of vegetables, fiber, and whole grains, excessive consumption of salt, sugar, and ultra-processed foods, and reduced thirst perception leading to dehydration [34,35,36,37,38].

### 4.2. Dietary Behaviors and Nutritional Risks

Although 50% of participants rated their health as “good”, they reported an average of 3.1 chronic conditions per person. The most common diagnoses were elevated cholesterol (67.7%), hypertension (53.1%), and obesity (29.2%), consistent with national and international studies [36,37,38,39]. Cardiovascular diseases remain the leading global cause of death, and their development is closely linked to modifiable lifestyle factors such as diet, physical inactivity, obesity, and tobacco and alcohol use [40].

In our study, 57.3% of respondents were on antihypertensive medication, and 52.1% were being treated for high cholesterol. A total of 62.5% of participants had a normal BMI; values between 23.0 and 29.9 are considered optimal for older adults. The so-called “obesity paradox”—wherein higher BMI is associated with lower mortality—has prompted debate about BMI’s utility in geriatrics [41]. Nevertheless, BMI remains relevant in diagnosing sarcopenia, which is linked to nutritional deficiencies and poor muscle function. Undernutrition was found in 9.7% of women, excluding the 71–80 age group.

A 2022–2023 study by Gajda et al. [19] demonstrated that unhealthy dietary patterns, such as the frequent consumption of white bread, pasta, butter, and processed meats, increase the risk of sarcopenia and frailty. Age-related sensory impairments further contribute to poor intake and nutritional risk. Despite a weak correlation (r = 0.3) between BMI and the number of diagnosed diseases, the association between excess weight and chronic conditions is well established. Globally, the disease burden related to elevated BMI has more than doubled over the past three decades [42,43].

Excess weight increases the risk of hypertension, cardiovascular diseases, type 2 diabetes, obstructive sleep apnea, and certain cancers. Obesity is officially classified as a disease by the WHO under ICD code E66 [44]. These findings align with conclusions from Larsson et al. [45], who, in a systematic review and Mendelian randomization meta-analysis, confirmed the causal role of high BMI in the development of multiple chronic conditions. Genetically determined higher BMI was found to increase the risk of type 2 diabetes, 14 cardiovascular diseases, asthma, chronic obstructive pulmonary disease, and 5 gastrointestinal diseases. The study also identified associations between elevated BMI and three musculoskeletal disorders, multiple sclerosis, and various cancers, including those of the gastrointestinal tract, uterus, kidney, and bladder.

These results reflect a concerning trend of dietary inconsistency and low adherence to medically indicated diets among older adults. Despite the widespread presence of chronic conditions in this population, only 12.5% reported following therapeutic or structured dietary plans. The predominance of three meals per day is consistent with the findings by Tańska et al. [22], but regularity remains limited. Only 18.8% consumed meals at consistent intervals, which may affect metabolic stability and appetite regulation. The high prevalence of eating at home may offer opportunities for improving dietary structure, but it also raises concerns about the quality of food choices, especially when coupled with frequent snacking. The preference for sweet snacks and the relatively low intake of vegetables or unsweetened options suggest dietary patterns with low nutritional density. These patterns echo observations by Śmidowicz et al. [37], who similarly found a tendency toward calorie-dense but nutrient-poor snack choices in older populations. Given that snacking may either supplement or displace balanced meals, its qualitative aspects deserve further attention in the context of healthy aging.

### 4.3. Diet Quality as a Predictor of Diet-Related Diseases

Our analysis using the pro-healthy diet index (pHDI-10) reveals a low average score (2.62), indicating insufficient adherence to health-promoting dietary patterns. Most participants reported a low intake of whole grains, legumes, fish, vegetables, and fruits. These results align with previous studies highlighting widespread deficiencies in pro-health dietary habits among Polish adults and seniors [46,47,48,49].

Whole-grain consumption has been shown to reduce cardiovascular risk, as demonstrated in the Iowa Women’s Health Study, where higher intake was associated with 18–30% lower mortality from heart conditions. Whole grains also support gut microbiota balance, benefiting immune and metabolic functions. The low intake of legumes and fish, reported by over 70% of participants, further weakens dietary quality and disease prevention.

Poor-quality diets—high in salt, sugar, and saturated fats and low in plant-based foods—significantly contribute to cardiovascular mortality. Petersen et al. [7] linked such diets to 53% of cardiovascular deaths and 58% of related disability. Even modest improvements in dietary quality can lower disease risk and extend lifespan. Public health strategies should clearly communicate to older adults that diet quality directly impacts aging and chronic disease development. Many seniors misattribute health decline solely to aging, overlooking the role of modifiable dietary factors.

The National Center for Nutrition Education recommends that older adults consume at least three large glasses of milk daily or substitute with fermented dairy products, which supply protein, calcium, magnesium, potassium, B vitamins, and vitamin D [50]. Charzewska [26] notes a trend among seniors to reduce dairy intake, partly due to limited awareness of its benefits, especially of fermented options. Milk and dairy intolerance, often due to lactose intolerance, allergies, or age-related digestive changes, may further discourage consumption.

Guantario et al. [27] suggest that milk, particularly the A2/A2 beta-casein variant, may help counteract age-related gut health deterioration. Moreover, in the context of prevalent overweight and obesity among older adults, Visioli et al. [28] indicate that milk proteins may aid in fat mass reduction, especially by decreasing visceral fat, as shown in studies involving healthy and overweight individuals, including those with diabetes.

### 4.4. Effectiveness and Reach of Nutritional Education

Supporting healthy aging requires integrated strategies emphasizing nutrition education, regular physical activity, and psychosocial support. Education should address age-specific challenges, such as appetite loss, chewing difficulties, sensory decline, and fluid intake. Programs should encourage aerobic, resistance, and weight-bearing exercises adapted to individual capacity. Although 80.2% of respondents believed diet should change with age, only 39.6% considered their knowledge sufficient. Barriers included entrenched habits (37.5%), limited access to free services (24%), and insufficient knowledge (21.9%) [7]. The Academy of Nutrition and Dietetics highlights that healthy eating reduces the risk of hypertension, heart disease, diabetes, obesity, and osteoporosis, particularly in seniors [11,51].

Education should be delivered by professionals trained in geriatric nutrition, using clear materials, large fonts, and accessible language. Despite this, 89.6% of respondents had not consulted a dietitian in the past year. Śmidowicz et al. [37] emphasize the need for educational efforts starting from age 40. Over half of the respondents acknowledged that nutrition education protects health, and nearly 90% believed it improves well-being. Nutrition education is a key public health tool to prevent under- and overnutrition, reduce health inequalities, and promote behavioral change [13,51]. Digital formats—like online workshops and eBooks—can enhance the reach, particularly for tech-savvy seniors (72.9% of respondents use computers or tablets). However, older participants often prefer traditional formats, and interventions should be tailored accordingly.

International programs confirm the effectiveness of tailored strategies. Canadian, Spanish, and Australian pilot studies showed that dietary workshops improved diet quality and motivation to cook [12,52,53,54]. Web-based tools also enhance dietary knowledge and self-efficacy [55].

Although nutrition education requires an initial investment, long-term savings are significant. The U.S. “Food is Medicine” program saved USD 13.6 billion in its first year and is projected to save USD 185.1 billion over a decade, preventing 1.6 million hospitalizations and gaining 260,000 QALYs [52]. In Poland, 2023 healthcare expenditures totaled PLN 155.9 billion, with 55.6% allocated to individuals over 60. Medication reimbursements for this group accounted for 65% of the total drug budget [5]. Investment in preventive strategies like nutrition education can ease the financial burden on healthcare systems.

Commensality—eating together—supports diet quality and emotional health by fostering social connection and reducing loneliness [56,57,58,59]. Mobility barriers, such as limited transport and physical constraints, must also be addressed to ensure equitable access to educational programs [54,55]. Effective nutrition education improves knowledge retention and fosters behavior change, even among isolated or cognitively impaired seniors. Its benefits extend beyond individuals, positively influencing communities and healthcare systems through a “domino effect”.

Nutrition education is a scientifically validated strategy for promoting health in older adults. Its implementation within primary care and public health frameworks can support sustainable, prevention-focused healthcare for aging populations.

## 5. Study Limitation

Although our study employed a short-term intervention with a modest sample, it aligns with findings from other research suggesting that even brief, well-structured education programs can improve nutrition knowledge and trigger behavior change, particularly when tailored to the cognitive and functional capacities of older adults [12,13,51]. However, several limitations must be acknowledged in light of the broader literature on geriatric nutrition interventions.

First, the use of a non-probability convenience sample (n = 151) recruited primarily from community centers and social care institutions limits the generalizability of our findings. As noted in prior studies [12,36,46], such sampling methods may exclude more isolated, frail, or digitally excluded seniors, who are often at a higher nutritional risk. Additionally, the predominance of women and concentration in specific age subgroups further constrain representativeness.

Second, the reliance on self-reported data—such as body weight, height, physical activity, and dietary intake—introduces known biases, including social desirability and recall errors. This is a recurring challenge in nutrition research among older adults, particularly given cognitive variability and polypharmacy [5,13]. Although we provided pre-survey instructions to improve comprehension, subjective responses may still limit internal validity.

Third, the cross-sectional design captures a single time point and does not allow for causal inferences regarding the relationship between nutrition education and health behavior change. The effectiveness and sustainability of nutrition interventions are best captured using longitudinal or randomized controlled designs, which are commonly used in larger-scale studies [13,50,55].

Fourth, while the study evaluated a wide array of lifestyle, nutritional, and health indicators, important contextual variables—such as economic hardship, access to fresh food, social support, and physical or sensory impairments—were not comprehensively addressed. These are recognized in the literature as significant social determinants of dietary behavior in older adults [14,38,54].

Fifth, the potential for selection bias must be acknowledged, as our recruitment strategy may have favored more socially engaged or health-conscious individuals (e.g., those participating in senior clubs or local education programs). Such individuals may already possess greater health literacy, limiting the applicability of findings to marginalized groups [55].

Finally, objective health indicators (e.g., biochemical markers, muscle mass, and nutrient levels) were not collected. While the use of self-assessment tools and diet indices (pHDI-10 and nHDI-14) is validated [18], combining them with clinical parameters—as recommended in comprehensive geriatric nutritional assessments—would enhance robustness and comparability with other studies [7,24,39].

Taken together, these limitations underscore the exploratory nature of this pilot study. Nevertheless, the findings provide meaningful insights into the dietary behaviors, education gaps, and public health potential of community-based interventions for Polish seniors. To address these gaps, future research should

(1) Include larger, probabilistically selected samples;
(2) Apply mixed-method or longitudinal approaches;
(3) Incorporate clinical and functional health measures;
(4) Target underrepresented groups, including rural and socially isolated individuals.

These steps would align with best practices in geriatric nutrition research and enhance the translation of educational interventions into evidence-based public health strategies.

## 6. Conclusions

This pilot study demonstrates that even short, structured nutrition education can enhance dietary awareness among older adults and potentially lead to positive behavior change. The findings confirm poor diet quality in this demographic, along with a high prevalence of chronic conditions, such as hypercholesterolemia, hypertension, and obesity. A weak but significant correlation between BMI and the number of diagnosed diseases underscores the relevance of dietary and lifestyle interventions. Most participants perceived education as a form of health protection and expressed readiness to share gained knowledge, highlighting the potential “domino effect” in community-based health promotion. Despite the study’s limitations, including its cross-sectional design and small sample size, the results support the integration of tailored nutrition education into primary care and geriatric outreach. Nonetheless, the findings provide meaningful insights into the dietary behaviors and educational needs of older adults in Poland and may serve as a valuable foundation for future larger-scale interventions. Future longitudinal research is needed to evaluate the sustainability of dietary improvements, the long-term health impact of education, and the effectiveness of inclusive models that address social and mobility barriers to healthy aging.

Most participants perceived education as a form of health protection and expressed readiness to share gained knowledge, highlighting the potential “domino effect” in community-based health promotion; however, this was explored subjectively and not objectively measured.

## Figures and Tables

**Table 1:** Diet quality index profiles with the proportion of study participants characterized by a given profile.

Profile Number	Type of Indicator	Quality Profiles	Participation of Participants (*N* = 151)	
			*N*	%
1	Low pHDI-10 and low nHDI-14	L-QI ^†^	90	60
2	Moderate pHDI-10 and low nHDI-14	M-QI ^††^	45	30
3	High pHDI-10 and low nHDI-14	H-QI ^†††^	16	11

^†^ “low diet quality index”; ^††^ “medium diet quality index”; ^†††^ “high diet quality index”.

**Table 2:** The general characteristics of the studied population.

Characteristics		*N* = 151
Gender	Female	91
	Male	60
Age	<60	0
	60–65	82
	66–70	40
	71–80	23
	>80	6
Place of residence	Village	23
	Small town	41
	City	87
Residential status	Live alone	35
	Live with a partner	56
	Live with children	24
	Multigenerational family	24
	Care institution	12
Employment activity	Yes	57
	No	94
Marital status	Single	15
	In a relationship	83
	Widowed	49
	Other	4
Education	Primary	3
	Vocational	12
	Secondary	60
	High	76
Body mass index (kg/m^2^)	<23.0	9
	23.0–29.9	86
	≥30.0	60

**Table 3:** Relationships between diet quality and anthropometric and sociodemographic data.

Characteristics		Profile of Diet Quality Index						*p*	Cramér’s V
		L-QI ^a^		M-QI ^b^		H-QI ^c^			
		*N* = 90	60%	*N* = 45	30%	*N* = 16	11%		
Gender	Female ^ab,bc,ac^	59	39	30	20	14	9	0.213	0.085
	Male ^ab,bc,ac^	31	21	15	10	2	1		
Age	60–65 ^ab,bc,ac^	25	17	45	30	12	8	<0.0001	0.451
	66–70 ^ab,bc,ac^	38	25	0	0	2	1		
	71–80 ^ab,bc,ac^	21	14	0	0	2	1		
	>80 ^ab,bc,ac^	6	4	0	0	0	0		
Place of residence	Village ^ab,bc,ac^	13	9	0	0	10	7	<0.0001	0.409
	Small town ^ab,bc,ac^	30	20	5	3	6	4		
	City ^ab,bc,ac^	47	31	40	26	0	0		
Residential status	Live alone ^ab,bc,ac^	35	23	0	0	0	0	<0.0001	0.411
	Live with A partner ^ab,bc,ac^	40	26	10	7	6	4		
	Live with children ^ab,bc,ac^	5	3	9	6	10	7		
	Multigenerational family ^ab,bc,ac^	18	12	6	4	0	0		
	Care institution ^ab,bc,ac^	12	8	0	0	0	0		
Employment activity	Yes ^ab,bc,ac^	48	32	9	6	0	0	<0.0001	0.392
	No ^ab,bc,ac^	42	28	36	24	16	11		
Marital status	Single ^ab,bc,ac^	14	9	1	1	0	0	0.017	0.177
	In a relationship ^ab,bc,ac^	49	32	22	15	12	8		
	Widowed ^ab,bc,ac^	24	16	21	14	4	3		
	Other ^ab,bc,ac^	4	3	0	0	0	0		
Education	Primary ^ab,bc,ac^	3	2	0	0	0	0	<0.0001	0.474
	Vocational ^ab,bc,ac^	10	7	2	1	0	0		
	Secondary ^ab,bc,ac^	57	38	0	0	3	2		
	High ^ab,bc,ac^	20	13	43	28	13	9		
BMI (kg/m^2^)	<23.0 ^ab,bc,ac^	3	2	6	4	0	0	0.0005	0229
	23.0–29.9 ^ab,bc,ac^	50	33	20	13	16	11		
	≥30.0 ^ab,bc,ac^	41	27	19	13	0	0		

^a^, L-QI; ^b^, M-QI; ^c^, H-QI; ^ab^, ^bc^, ^ac^, significant differences between profiles of diet quality indicators and characteristics of the study population; chi-square test; *p* < 0.05.

## Data Availability

The original data presented in this study are available from the corresponding author.

## Supplementary Materials

The following supporting information can be downloaded at: https://www.mdpi.com/article/10.3390/nu17132103/s1, Table S1. Questions and answers; Figure S1. Declared frequency of alcohol consumption; Figure S2. Declared frequency of choosing a place to eat meals; Figure S3. Declared frequency of consuming food products from different groups. Declared frequency of alcohol consumption; Figure S4. Declared frequency of consuming animal products; Figure S5. Declared frequency of consuming plant products.
