# Supplementary material for: Nutrition Education Among Community-Dwelling Polish Seniors—A Pilot Study of Diet Quality, Health Status, and Public Health Interventions
Source: Nutrients. 2025 Jun 25;17(13):2103. doi: 10.3390/nu17132103 (PMC12251276; doi:10.3390/nu17132103)
Supplement: Supplementary file 1 [file nutrients-17-02103-s001.zip › nutrients-3691789-supplementary.pdf]

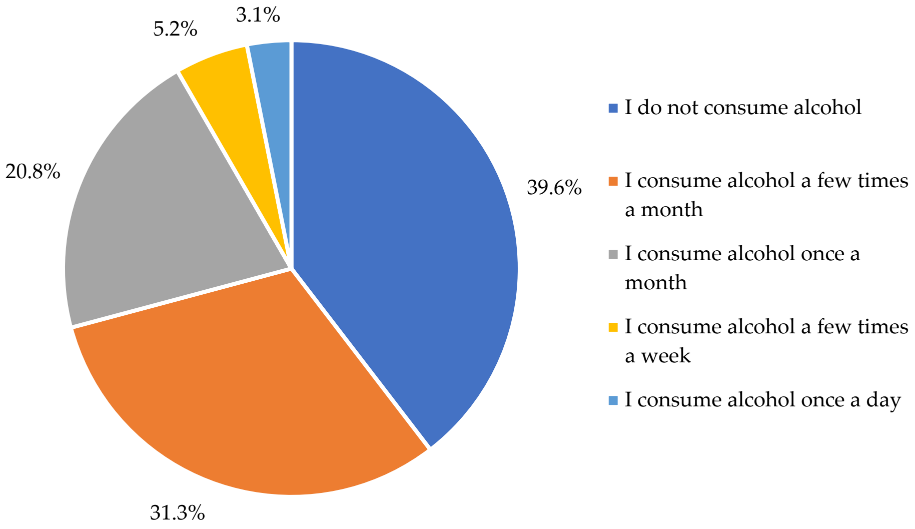

Figure S1. Declared frequency of alcohol consumption

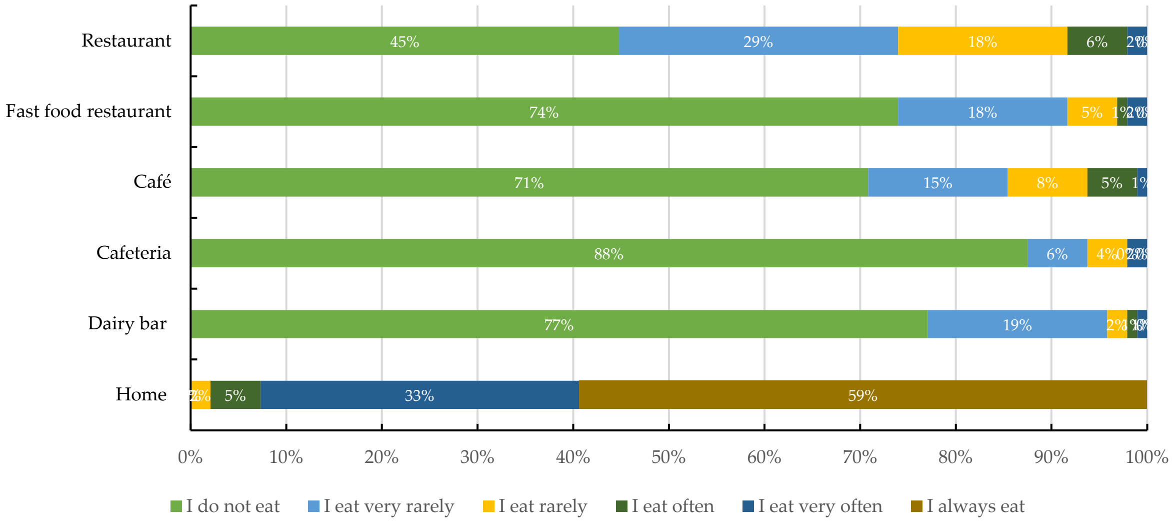

Figure S2. Declared frequency of choosing a place to eat meals

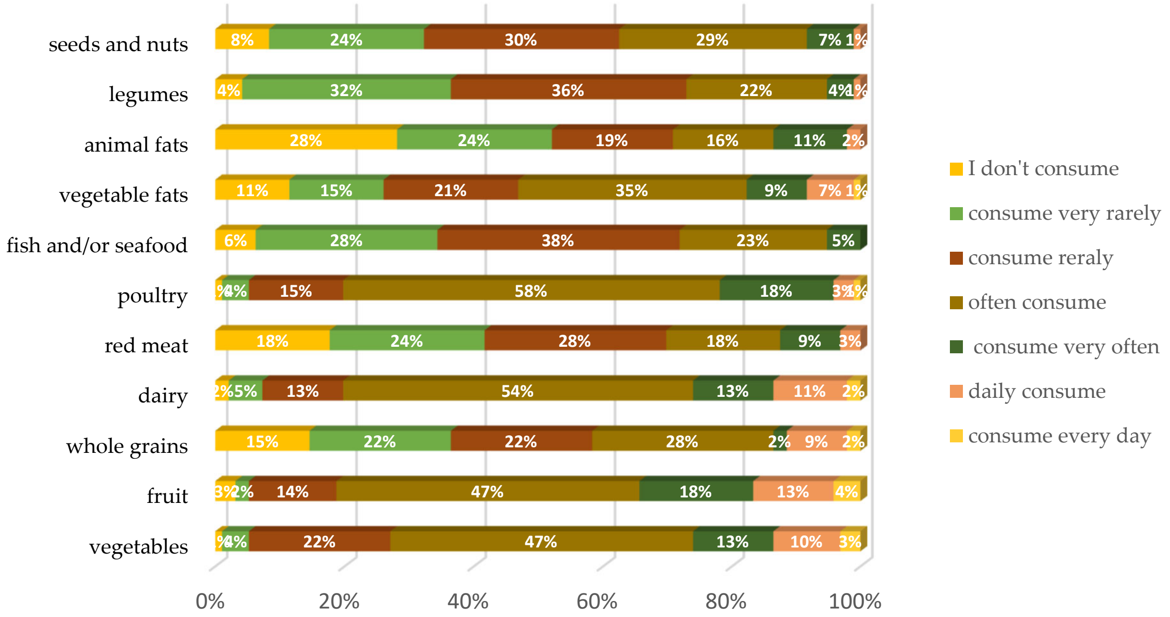

Figure S3. Declared frequency of consuming food products from different groups

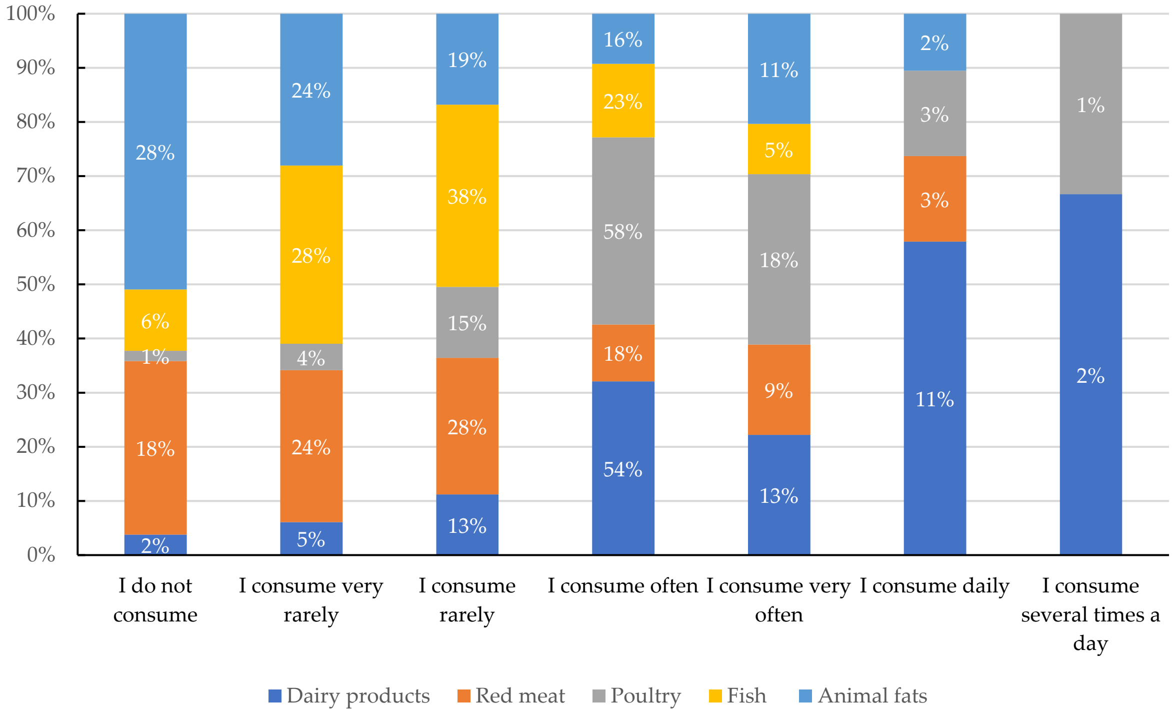

Figure S4. Declared frequency of consuming animal products

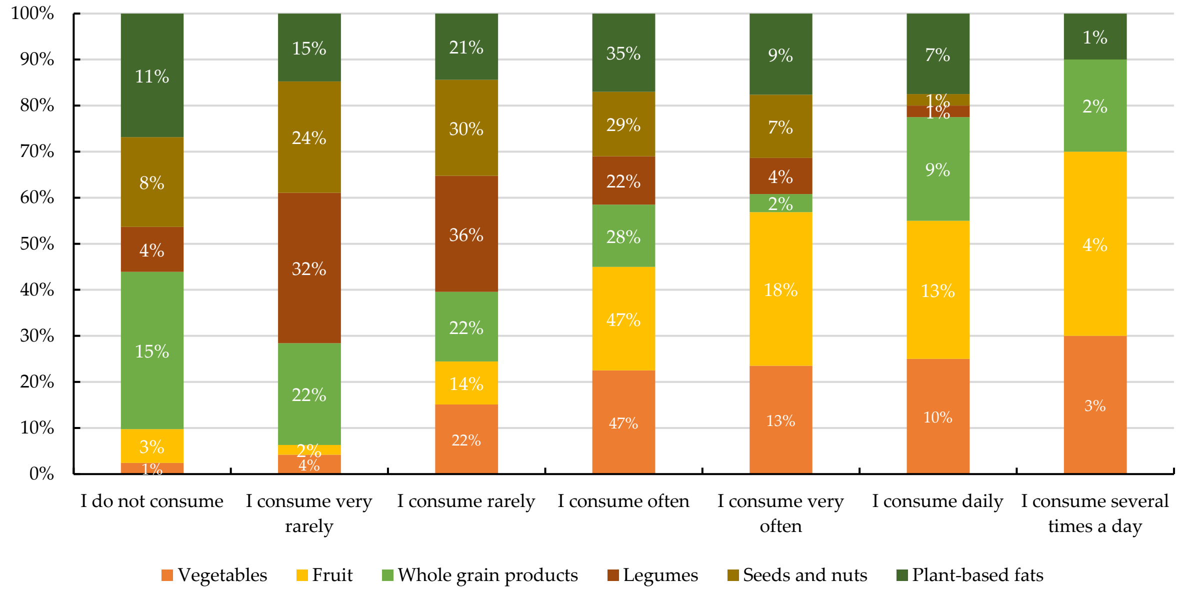

Figure S5. Declared frequency of consuming plant products

Table S1. Questions and answer

**Questions and answer**	**100% (*N=151*)**
How do you asses the situation of your household	
very modern	2.1%
modern	7.3%
average	36.5%
good	39.6%
very good	14.6%
Are you a member of the Seniors' Club?	
yes	26.0%
no	74.0%
Do you use the internet and a computer and/or a tablet?	
yes	72.9%
no	27.1%
"What, in your opinion, makes it difficult to engage in regular physical activity?	
Reluctance to leave the house	11.5%
Lack of motivation	26.0%
Lack of time	9.4%
Current health condition	30.2%
Financial issues related to engaging in physical activity, such as the costs of memberships, entry fees for organized classes, swimming pool, etc.	8.3%
Lack of or limited access to free classes for seniors	14.6%
How do you assess the quality of your sleep?	

very good	8.3%
good	44.8%
neither good, nor bad	33.3%
bad	13.5%
very bad	0.0%
Are you a person with a disability certificate?	
yes - large extent	4.2%
yes - moderate extent	10.4%
yes - slight extent	9.4%
no	76.0%
What is your subjective perception of aging?	
I feel very old	2.1%
I feel old	10.4%
I neither feel old nor young	70.8%
I feel young	16.7%
I feel very young	0.0%
Do you experience stress and/or nervousness related to aging?	
I don't really feel it	17.7%
I don't feel it	36.5%
I have no opinion	16.7%
Yes, I feel it	27.1%
Yes, I feel it strongly	2.1%
How do you currently assess the state of your health?	
Very good	1.0%
Good	50.0%
Sufficient	38.5%
Bad	9.4%
Very bad	1.0%
Are you currently following any diet?	
yes	87.5%
no	12.5%
Please indicate the type of diet you are following	
Diabetic diet	41.7%
Easily digestible diet	33.3%
Intermittent fasting	16.7%
Meal-box diet	8.3%
How many meals do you eat daily?	
1 meal	0.0%
2 meals	4.2%
3 meals	51.0%
4 meals	30.2%
5 meals	13.5%
6 meals or more	1.0%

"Do you eat meals at regular times during the day?	
No	26.0%
Some meals I eat at regular times	55.2%
Yes, I eat all meals at regular times	18.8%
In your opinion, does the regularity of meal consumption affect overall health?	
Yes	68.8%
No	2.1%
I have no opinion	29.2%
In your opinion, does snacking between meals affect overall health?	
Yes	55.2%
No	9.4%
I have no opinion	35.4%
Do you smoke?	
Yes	8.3%
No	91.7%
Do you sweeten beverages such as coffee, tea, compote, cocoa?	
I don't sweeten	54.2%
Yes, I add 1 teaspoon of sugar or honey	33.3%
Yes, I add 2 or more teaspoons of sugar or honey	10.4%
Yes, I use sugar substitutes such as sweeteners	2.1%
In your subjective opinion, what is your daily salt intake?	
Much less than 1 teaspoon of salt	22.9%
Less than 1 teaspoon	31.3%
More than 1 teaspoon of salt	27.1%
1 teaspoon of salt	16.7%
Much more than 1 teaspoon of salt	2.1%
In your opinion, does the daily addition of salt to products and/or meals affect overall health?	
Very little impact	5.2%
Small impact	11.5%
I have no opinion	34.4%
Large impact	38.5%
Very large impact	10.4%
How many eggs do you consume per week?	
I don't consume eggs	1.0%
1-2 per week	29.2%
3-4 per week	40.6%
5-6 per week	18.8%
7 or more per week	10.4%

What dairy products do you consume most frequently?	
fat-free	4.2%
reduced-fat	21.9%
semi-fat	58.3%
full-fat	15.6%
Yes	88.5%
No	11.5%
What form of preparing fish for consumption do you prefer?	
raw	2.0%
boiled	6.1%
stewed	10.1%
baked	15.6%
grilled	12.6%
fried	17.6%
smoked	18.2%
canned in oil/sauces	10.3%
fish spreads	4.7%
salads - ready-made products	2.8%
Do you consume pickled vegetables and/or fruits?	
I don't consume	2.1%
yes, I consume occasionally - every few months	17.7%
yes, I consume once a week	33.3%
yes, I consume a few times a week	42.7%
yes, I consume once a day	2.1%
yes, I consume several times a day	2.1%
Which legume seeds do you prefer?	
beans	32.1%
peas	24.4%
lentils	9.5%
chickpeas	10.7%
broad beans	21.8%
soybeans	1.5%
others	0.0%
What type of thermal processing for preparing dishes do you or the person preparing them prefer?	
raw - without the need for thermal processing	
I do not use	53.2%
I use once a year	2.1%
I use a few times a year	7.4%
I use once a month	6.4%
I use a few times a month	9.6%

I use once a week	10.6%
I use a few times a week	6.4%
I use once a day	3.2%
I use a few times a day.	1.1%
Steaming/blanching	
I do not use	44.2%
I use once a year	3.2%
I use a few times a year	14.7%
I use once a month	5.3%
I use a few times a month	15.8%
I use once a week	9.5%
I use a few times a week	4.2%
I use once a day	1.1%
I use a few times a day.	2.1%
Cooking	
I do not use	7.2%
I use once a year	2.1%
I use a few times a year	0.0%
I use once a month	0.0%
I use a few times a month	17.5%
I use once a week	13.4%
I use a few times a week	43.3%
I use once a day	10.3%
I use a few times a day.	6.2%
Braising	
I do not use	9.5%
I use once a year	0.0%
I use a few times a year	5.3%
I use once a month	8.4%
I use a few times a month	17.9%
I use once a week	21.1%
I use a few times a week	33.7%
I use once a day	4.2%
I use a few times a day.	0.0%
Grilling	
I do not use	36.2%
I use once a year	5.3%
I use a few times a year	27.7%
I use once a month	9.6%
I use a few times a month	10.6%
I use once a week	6.4%
I use a few times a week	3.2%
I use once a day	1.1%
I use a few times a day.	0.0%

Baking	
I do not use	14.9%
I use once a year	2.0%
I use a few times a year	6.9%
I use once a month	8.9%
I use a few times a month	22.8%
I use once a week	22.8%
I use a few times a week	20.8%
I use once a day	1.0%
I use a few times a day.	0.0%
Frying with a small amount of fat	
I do not use	12.5%
I use once a year	4.2%
I use a few times a year	8.3%
I use once a month	9.4%
I use a few times a month	22.9%
I use once a week	22.9%
I use a few times a week	17.7%
I use once a day	2.1%
I use a few times a day.	0.0%
Deep-fat frying	
I do not use	66.0%
I use once a year	2.1%
I use a few times a year	8.5%
I use once a month	9.6%
I use a few times a month	7.4%
I use once a week	6.4%
I use a few times a week	0.0%
I use once a day	0.0%
I use a few times a day.	0.0%
Do you consume ready-made products, such as powdered soups, instant meals, ready meals?	
I don't consume	81.3%
I consume a few times a month	15.6%
I consume a few times a week	3.1%
I consume daily	0.0%
I consume several times a day	0.0%
How often do you consume fast food?	
I don't consume	43.8%
I consume once a year	12.5%
I consume a few times a year	36.5%
I consume once a month	3.1%
I consume a few times a month	4.2%
I consume once a week	0.0%

I consume a few times a week	0.0%
I consume daily	0.0%
Do you take dietary supplements?	
Yes	50.0%
No	50.0%
What motivated you to start taking dietary supplements?	
Making the decision to improve your current state of health	32.4%
doctor's recommendations	31.1%
Pharmacist's recommendation	6.8%
recommendation of a dietitian	8.1%
Recommendation from family, friends, neighbors	13.5%
advertising - TV and/or press	8.1%
What dietary supplements do you take?	
Magnesium	
I do not take them	37.5%
I take them during the autumn-winter period	10.4%
I take them a few times a month	5.2%
take them a few times a week	10.4%
I take them daily	36.5%
calcium	
I do not take them	63.5%
I take them during the autumn-winter period	5.2%
I take them a few times a month	6.3%
take them a few times a week	5.2%
I take them daily	19.8%
Vitamin C	
I do not take them	41.7%
I take them during the autumn-winter period	21.9%
I take them a few times a month	7.3%
take them a few times a week	7.3%
I take them daily	21.9%
Sodium + potassium	
I do not take them	66.7%
I take them during the autumn-winter period	3.1%
I take them a few times a month	6.3%
take them a few times a week	7.3%
I take them daily	16.7%
Iron	
I do not take them	89.6%
I take them during the autumn-winter period	1.0%
I take them a few times a month	5.2%
take them a few times a week	0.0%

I take them daily	4.2%
Vitamin B12	
I do not take them	58.3%
I take them during the autumn-winter period	8.3%
I take them a few times a month	10.4%
take them a few times a week	7.3%
I take them daily	15.6%
Folic acid	
I do not take them	91.7%
I take them during the autumn-winter period	5.2%
I take them a few times a month	1.0%
take them a few times a week	0.0%
I take them daily	2.1%
Electrolytes	
I do not take them	75.0%
I take them during the autumn-winter period	6.3%
I take them a few times a month	9.4%
take them a few times a week	5.2%
I take them daily	4.2%
Multivitamin preparations	
I do not take them	68.8%
I take them during the autumn-winter period	10.4%
I take them a few times a month	3.1%
take them a few times a week	7.3%
I take them daily	10.4%
Cod liver oil and/or omega-3 fatty acids	
I do not take them	78.1%
I take them during the autumn-winter period	10.4%
I take them a few times a month	1.0%
take them a few times a week	3.1%
I take them daily	7.3%
Fiber	
I do not take them	81.3%
I take them during the autumn-winter period	1.0%
I take them a few times a month	6.3%
take them a few times a week	6.3%
I take them daily	5.2%
Herbal preparations supporting bowel movements	
I do not take them	91.7%
I take them during the autumn-winter period	0.0%
I take them a few times a month	4.2%
take them a few times a week	4.2%
I take them daily	0.0%
Herbal sleep aids	

I do not take them	85.4%
I take them during the autumn-winter period	0.0%
I take them a few times a month	8.3%
take them a few times a week	3.1%
I take them daily	3.1%
Herbal preparations lowering lipid levels	
I do not take them	91.7%
I take them during the autumn-winter period	0.0%
I take them a few times a month	3.1%
take them a few times a week	1.0%
I take them daily	4.2%
Herbal preparations lowering blood sugar levels	
I do not take them	91.7%
I take them during the autumn-winter period	0.0%
I take them a few times a month	2.1%
take them a few times a week	1.0%
I take them daily	5.2%
In your opinion, should the way of eating change with age?	
Yes	80.2%
No	1.0%
I have no opinion	18.8%
How do you assess your current eating habits?	
Very bad	2.1%
Bad	5.2%
Neither bad nor good	46.9%
Good	44.8%
Very good	1.0%
How do you assess your knowledge about healthy nutrition?	
Very good	4.2%
Good	29.2%
Satisfactory	39.6%
Little	21.9%
Very little	5.2%
Have you sought dietary advice in the past year?	
Yes	10.4%
No	89.6%
Do you think that nutrition education for seniors is a form of health protection?	
I strongly disagree	2.1%
I disagree	1.0%
I have no opinion	15.6%
I agree	52.1%

I strongly agree	29.2%
Fear of introducing dietary changes	
Current dietary habits/customs	37.5%
Fear of introducing dietary changes	4.2%
Insufficient knowledge in the area of nutrition education	21.9%
Lack of knowledge in the field of nutrition education	12.5%
Difficulty in access to	24.0%
Do you think that nutrition education for seniors has an impact on improving overall health?	
Yes	89.6%
No	0.0%
I have no opinion	10.4%
